# Evaporation Kinetics and Final Particle Morphology of Multicomponent Salt Solution Droplets

**DOI:** 10.1021/acs.jpca.4c07439

**Published:** 2025-01-11

**Authors:** Barnaby E. A. Miles, Emily Winter, Shaira Mirembe, Daniel Hardy, Lukesh K. Mahato, Rachael E. H. Miles, Jonathan P. Reid

**Affiliations:** School of Chemistry, University of Bristol, Bristol BS8 1TS, U.K.

**Keywords:** aerosol, drying, crystallization, single-droplet, chloride, sulfate

## Abstract

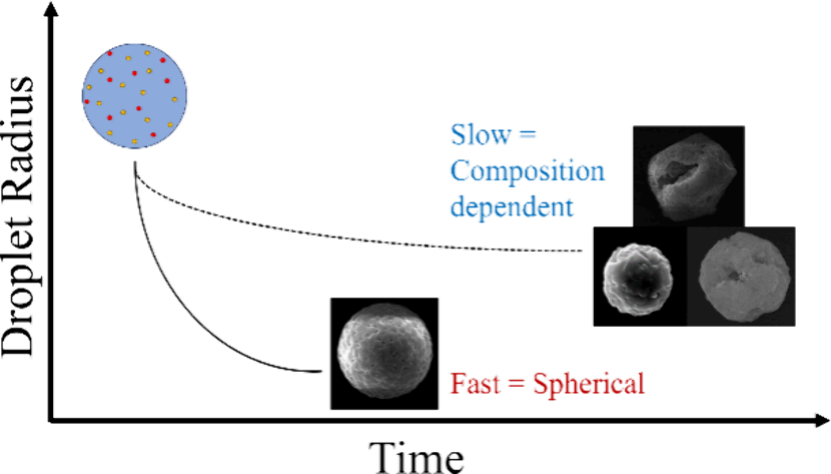

In both nature and industry, aerosol droplets contain complex mixtures of solutes, which in many cases include multiple inorganic components. Understanding the drying kinetics of these droplets and the impact on resultant particle morphology is essential for a variety of applications including improving inhalable drugs, mitigating disease transmission, and developing more accurate climate models. However, the previous literature has only focused on the relationship between drying kinetics and particle morphology for aerosol droplets containing a single nonvolatile component. Here we investigate the drying kinetics of NaCl-(NH_4_)_2_SO_4_, NaCl-NH_4_NO_3_, and NaCl-CaCl_2_ mixed salt aqueous aerosol droplets (25−35 μm radius) and the resulting morphology and composition of the dried microparticles. A comparative kinetics electrodynamic balance was used to measure evaporation profiles for each mixed salt aerosol at a range of relative humidities (RH) (0−50% RH); measurements of the evaporation kinetics are shown to be consistent with predictions from the “Single Aerosol Drying Kinetics and Trajectories” model. Populations of the mixed salt droplets were dried in a falling droplet column under different RH conditions and imaged using scanning electron microscopy to observe the impact of the drying kinetics on the morphology. Energy dispersive spectroscopy was used in tandem to obtain atomic maps and view the impact of drying kinetics on the composition of the resultant particles. It has been shown that the relationship between drying kinetics and dry particle morphology in mixed salt solution droplets is compositionally dependent and determined by the predominant salts that crystallize (i.e., (NH_4_)_2_SO_4_, Na_2_SO_4_, or NaCl). The degree of homogeneity in composition throughout the particle microstructure is dependent on the drying rate.

## 1 Introduction

Understanding the mass transport rates of water from and to of aerosol droplets is crucial to improving numerous industrial processes,^1−3^ understanding cloud droplet size distributions in the atmosphere,^4^ and deepening knowledge on airborne disease transmission.^5,6^ In particular, an understanding of the influence of drying kinetics on the morphology of a resultant dried particle is often lacking. Controlling the morphology of a pharmaceutical product in spray drying is desirable to obtain an aerodynamic diameter and solubility that optimize deposition in the lung and effective transition through the lung lining.^7−9^ Furthermore, as properties that make aerosols desirable for pharmaceuticals also make them effective carriers for viruses,^10−12^ linking respiratory aerosol properties to the drying kinetics and particle morphology can inform the choice of effective mitigation strategies for disease transmission (e.g., physical distancing).^13^ Morphological control in spray drying is also crucial when developing food powders, where spherical particles with minimal surface irregularities are preferred.^14^ Particles with irregular shapes have a detrimental effect on the powder flow properties, affecting the dosing, mixing, and coating of the product.^15,16^ Understanding the impact of the drying conditions on the resultant powder properties can help increase the spray dryer efficiency and product quality.^17^

There have been a significant number of investigations into the drying kinetics of aerosol droplets containing single involatile components^18,19^ and how these drying kinetics can affect the morphology of the resultant microparticles.^20−23^ This work has shown that for aerosol droplets containing either soluble or insoluble components, the drying kinetics influences the resultant morphology through a relationship between the evaporation rate and the diffusion of constituents within the droplet. This relationship can be represented using the dimensionless Peclet number (*Pe*):^24^
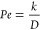
1where *k* is the evaporation rate and *D* is the diffusion constant of the nonvolatile component (e.g., solute ions, insoluble nanoparticles) within the droplet. The evaporation rate here is obtained from the “radius-square law”.^25^

When the evaporation rate is much larger than the internal mass transport rate (i.e., *Pe* ≫ 1), water evaporates faster than the nonvolatile components can redistribute within the droplet. This results in a concentration gradient of the nonvolatile component within the droplet, with enrichment of the component at the droplet surface. For droplets containing soluble components (e.g., inorganic salts), this increased surface enrichment increases the likelihood of a critical supersaturation of solute occurring at the droplet surface and then a nucleation event. Droplets containing inorganic salts that dry under conditions of low relative humidity (RH) or high temperature are therefore more spherical in shape due to the higher number of nucleation sites during the drying process.^20,26^ Additionally, surface enrichment means a lower nonvolatile concentration at the center of the droplet. Therefore, the resulting particles have increasingly large cavities in their center as the evaporation rate increases.^21,22^ If the evaporation rate is similar to the internal diffusion rate of components within the droplet, then they are able to redistribute during the evaporation process, resulting in an isotropic reduction in the droplet size. For droplets containing soluble components, this yields denser resultant particles that are more likely to have a single crystal structure.^20,26^ Hardy et al. (2021) confirmed this trend with aqueous NaCl droplets dried under different evaporation rates. At 0% RH, the particles had a spherical, multicrystal morphology, whereas at 40% RH, cubic, single-crystal particles were formed.^20^ Baldelli et al. (2016) investigated the drying kinetics of NaNO_3_ in spray dryers with results again supporting this surface enrichment mechanism. NaNO_3_ droplets dried at increasingly large temperatures resulted in spherical particles that increased in size as they had increasingly large cavities within, indicating an increase in droplet surface enrichment with evaporation rate.^27^

Although this relationship between drying kinetics and particle morphology has been established for droplets containing single nonvolatile components, a similar relationship has not yet been established for multicomponent nonvolatile systems, at least to the authors’ knowledge. The work by Baldelli and Vehring (2016) investigated the effect of crystallization time on the morphological properties of aqueous droplets containing mixes of NaNO_3_-KNO_3_.^28^ They found a preferential surface enrichment of the solute that reached supersaturation first and that both the initial composition and drying conditions affected the resultant particle density. Droplets that dried with a longer precipitation window resulted in less dense particles due to the formation of internal voids. This demonstrated control of multicomponent salt particle morphology using temperature and composition but was only shown for one system of salts.

There has been significant work on the changes in the efflorescence RH of solute components on addition of an extra nonvolatile component to an aerosol system.^29−33^ For mixtures of inorganic salts, a number of systems have been investigated, with both stepwise and single efflorescence events observed.^30,31^ Both processes are initiated by homogeneous nucleation of the most supersaturated component. In the stepwise mechanism, a eutonic mix of the salts then crystallizes at a mutual efflorescence relative humidity (MERH), which is lower than the efflorescence relative humidity (ERH) of either pure component.^30,34^ In the single-step efflorescence, the less saturated salt instantly crystallizes heterogeneously around the other salt crystal.^31,34^ The resulting mixed salt particles are usually observed to be a pure salt core surrounded by either a eutonic mix of salts or a shell of the other pure salt (seen in KCl/NaCl, KCl/KI, and (NH_4_)_2_SO_4_/NH_4_NO_3_).^29^

In this work, we report the effect of RH on the drying kinetics of single levitated droplets containing multiple inorganic salts using a comparative kinetics electrodynamic balance (CK-EDB) and relate the drying kinetics to the morphology of dried particles obtained from populations of droplets dried in a falling droplet column (FDC) by imaging them with scanning electron microscopy (SEM). Energy dispersive spectroscopy (EDS) is used in tandem with SEM imaging to obtain atomic maps of the resultant particles to check their composition and to observe any changes with drying kinetics. The Single Aerosol Drying Kinetics and Trajectories (SADKAT) model is used to validate the drying kinetic measurements obtained from the CK-EDB. This model has not previously been used for modeling multisalt droplet evaporation, but Tian et al. (2024) reported the successful validation of evaporation profiles for droplets containing complex components such as artificial saliva and Dulbecco’s modified Eagle’s medium.^11^Sections 3.1 and 3.2 investigate the effect of combining differing molar ratios of NaCl and (NH_4_)_2_SO_4_ salts in an aerosol droplet. Sections 3.3 and 3.4 then investigates the impact of adding NH_4_NO_3_ and CaCl_2_ (respectively) to a NaCl aerosol droplet.

## 2 Experimental Section

### 2.1 Salt Solution Preparation

Sodium chloride (NaCl; ≥99.5%), ammonium sulfate ((NH_4_)_2_SO_4_; ≥99.5%), and ammonium nitrate (NH_4_NO_3_; ≥99.5%) were purchased from Sigma-Aldrich (UK). Calcium chloride (anhydrous, CaCl_2_; ≥93.0%) was purchased from Honeywell Fluka (UK). Deionized water obtained from a HP 320 Type II Deionized Water System was used for all experiments. Three solutions of NaCl:(NH_4_)_2_SO_4_ were prepared with a NaCl mole fraction of 0.25, 0.50, and 0.75. Solutions of NaCl:NH_4_NO_3_ and NaCl:CaCl_2_, with NaCl mole fractions of 0.50 and 0.65, respectively, were also prepared. All solutions prepared had a solid mass fraction (MFS) of 0.02 with the exception of the NaCl:CaCl_2_ solution, which had a solid mass fraction of 0.1. For each 0.02 MFS sample, 1 g of the combined salt was weighed into a 100 mL beaker with 49 g of deionized water. The solution was placed in a Clifton Heated Timed Ultrasonic Bath and subjected to agitation by ultrasound for 3 min to ensure that the solutes had fully dissolved. For the 0.1 MFS sample, 2 g of the combined salt was dissolved in 18 g of deionized water.

### 2.2 Droplet Drying Measurements

The study of aerosol drying kinetics using the confinement and levitation of single droplets has been explored extensively in the literature.^35,36^ In this work, a comparative kinetics electrodynamic balance (CK-EDB) was used. This instrument levitates single charged aerosol droplets in an electric field between two cylindrical electrodes. The electrodes are positioned in a chamber that has controlled temperature and RH. The CK-EDB will only be discussed here briefly as it has been documented extensively in previous publications.^18,21,37−39^

A droplet-on-demand generator (MicroFab, MJ-ABP-01, orifice = 30 μm diameter) dispenses single aerosol droplets (initial radius = 25−35 μm, charge polarity ≈ −30 fC) into the stable region of the electric field in the CK-EDB trapping chamber. A 532 nm laser (Laser Quantum, Ventus continuous wave [CW]), aligned through the trapping chamber, illuminates the trapped droplet elastically scattering light that is collected with a CCD camera. The angular range of the scattered light collection is ∼24° centered at 45° to the propagation direction of the laser beam. Comparison of this scattered light intensity (the phase function) to the geometrical optics approximation (with corrections for changes in density and refractive index) allows for estimates of the variation in droplet radius with time.^39,40^ A 200 mL min^−1^ flow of nitrogen gas (20 °C, RH ∼0 to >90%) passes over the trapped droplet. Changes to the phase function allow for the detection of the efflorescence phase transition. When the droplet is spherical and homogeneous, it produces regularly spaced interference fringes in the angularly resolved scattering pattern. However, the phase transition to a dry salt particle causes a rapid loss in the sphericity of the aerosol producing irregular fringes and indicates the point at which efflorescence occurs.

### 2.3 Collection of Dried Microparticles

Populations of dried microparticles were produced by drying aqueous aerosol droplets in a falling droplet column (FDC). These resultant microparticles were then imaged using scanning electron microscopy (SEM) to investigate the morphology. This instrument has been detailed in the previous literature and so will only be briefly reviewed here.^20^ To ensure comparability with the CK-EDB drying kinetic measurements, a stream of uniform droplets with the same known composition and size (initial radius = 18−22 μm) was generated by a droplet-on-demand dispenser (Microfab, MJ-ABP-01, orifice = 30 μm diameter). These droplets were injected horizontally into the top of a 50 cm tall glass column so that they then fall vertically through the center of the column. A 532 nm vertically propagating laser was used to ensure that the droplets fall in the center of the column. This avoids the loss of the droplets/particles through collision with the column walls. The droplets dried as they fell, and a glass slide was positioned at the bottom of the glass column to collect the resultant microparticles. A 500 mL min^−1^ flow of nitrogen gas (20 °C, RH ∼0 to >90%) was passed through the column for all experiments. An RH probe and K-type thermocouple were used to monitor the conditions within the column continuously. The glass slides containing the salt microparticle samples were stored at 66 °C in a Genlab E3 drying cabinet to ensure that they remained dry. The slides were then attached to an aluminum stub and sputter-coated with a thin layer of carbon. The stub was placed in an SEM instrument (JSM-IT300, JEOL) with energy dispersive spectroscopy (EDS) capability, and images were recorded under ultrahigh vacuum at 15 kV and a working distance of 10.0 mm.

### 2.4 Modeling Evaporation Profiles for Data Validation

The drying kinetics of the aqueous salt droplets were simulated by using the Single Aerosol Drying Kinetics and Trajectories (SADKAT) model. This is a free-to-use and open-source program that is capable of calculating complete droplet trajectories and evaporation profiles. A brief review of the model has been given here as it has been discussed in previous publications.^26,41^

Previously, SADKAT has been shown to accurately model the evaporation and condensation processes for droplets with one volatile and one involatile component. In this work, we modeled the evaporation process in droplets with one volatile and two involatile components (aqueous droplets with two inorganic salts) by considering the multiple involatile components as a single combination of involatile ions. For example, a droplet of water, NaCl, and (NH_4_)_2_SO_4_ would be treated as a droplet of water and Na(NH_4_)_2_ClSO_4_, where the Na(NH_4_)_2_ClSO_4_ component has an assumed density of 1.871 g cm^−3^ and an *M*_r_ of 95.86 g mol^−1^. These density, molar mass, and refractive index parametrizations of the solution properties for the combined salts were calculated using the ideal mixing rule.^42^ The parametrization of water activity with mass fraction of solute (MFS) was calculated using the Extended-AIM Aerosol Thermodynamics Model (E-AIM).^43−45^ These insoluble component parametrizations can be found in Table S1. The input parameters required by SADKAT to model the evaporation profiles (RH, ambient temperature, droplet temperature, air flow rate, initial droplet diameter, and initial droplet velocity) were taken from the experimental measurements, and the values used for each model can be found Table S2. SADKAT assumes that all droplets are thermally and compositionally homogeneous and that they remain a solution; therefore, the model does not account for evaporation-induced surface enrichment or crystallization.

## 3 Results and Discussion

### 3.1 Effect of Drying Kinetics on the Final Morphology of Pure NaCl and (NH_4_)_2_SO_4_ Microparticles

**Figure 1:**
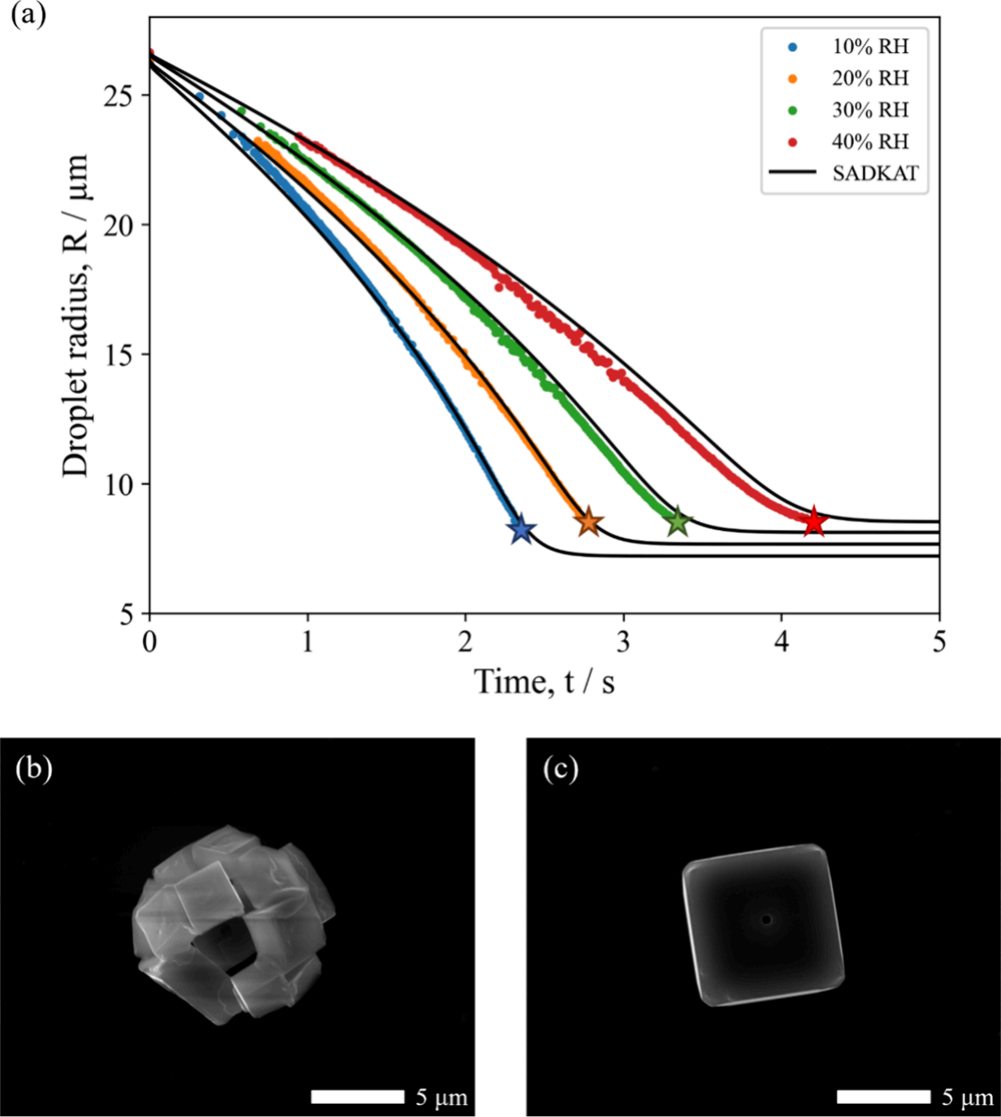
(a) Evaporation profiles for aqueous NaCl (0.02 MFS) droplets at varying RHs and 293.0 K compared with evaporation profiles modeled by SADKAT. Crystallization occurs at the time point indicated by a star, where the average *a*_w_ was calculated to be 0.381 ± 0.016 and the estimated dry particle size was 5.57 ± 0.04 μm. (b) SEM image of a NaCl microparticle dried at 0% RH, 294 K. (c) SEM image of a NaCl microparticle dried at 40% RH, 294 K.

**Figure 2:**
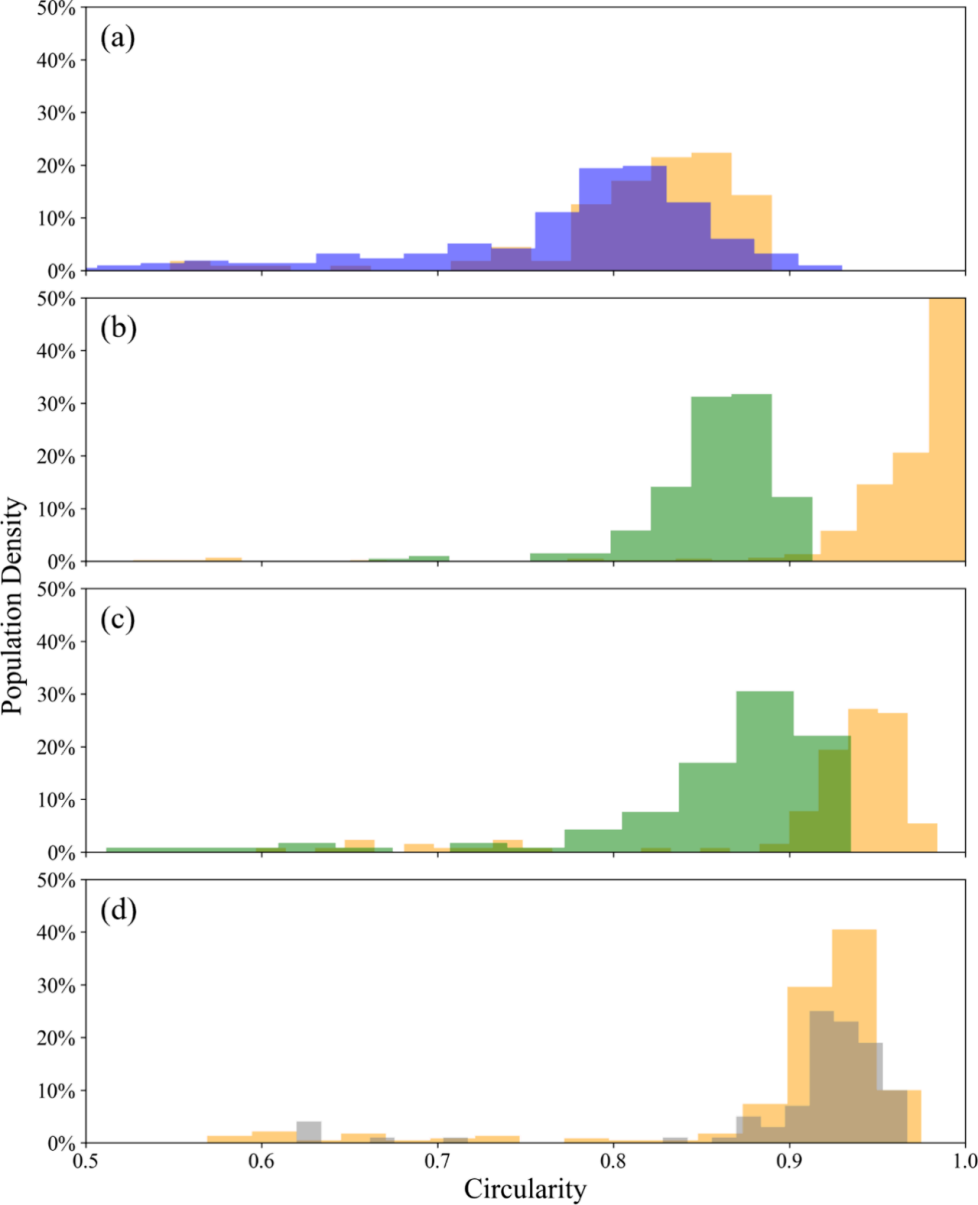
Degree of circularity for populations of dried particles at varying RHs. 0% RH = orange, 20% RH = green, 30% RH = gray, 40% RH = blue. (a) NaCl, (b) NaCl-(NH_4_)_2_SO_4_ (*X*_NaCl_ = 0.75), (c) NaCl-(NH_4_)_2_SO_4_ (*X*_NaCl_ = 0.50), and (d) (NH_4_)_2_SO_4_.

The drying kinetics of NaCl aerosol droplets and the impact on the morphology of the dried microparticles are well established.^18,20,26^ Therefore, to validate the experimental methods used in this work, a single-salt NaCl system was investigated to compare to the previous literature. The evaporation profiles (radius vs time) in Figure 1a show close agreement between the EDB measurements and SADKAT model at a range of RHs, and the SEM images of the pure NaCl microparticles (Figure 1b,c) show the same trend shown by Hardy et al. (2021).^20^ At high RHs, the particles have a cubic, single-crystal morphology, whereas at low RHs, the particles then have a circular, multicrystal morphology. This decrease in circularity with RH is demonstrated in Figure 2a, where the circularity of a particle was calculated from the SEM images using
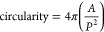
2

*A* is the area of the particle, and *P* is the perimeter of the particle. Both the area and perimeter were calculated using the ImageJ software.^46^ This plot supports the qualitative observation that an increased RH decreases the circularity of the particles. The difference in the dry sizes of the particles produced in the EDB versus the FDC (e.g., Figure 1) is expected given the difference in sizes of droplets generated for each technique (see Sections 2.2 and 2.3). This size difference is not expected to affect the drying behavior.

**Figure 3:**
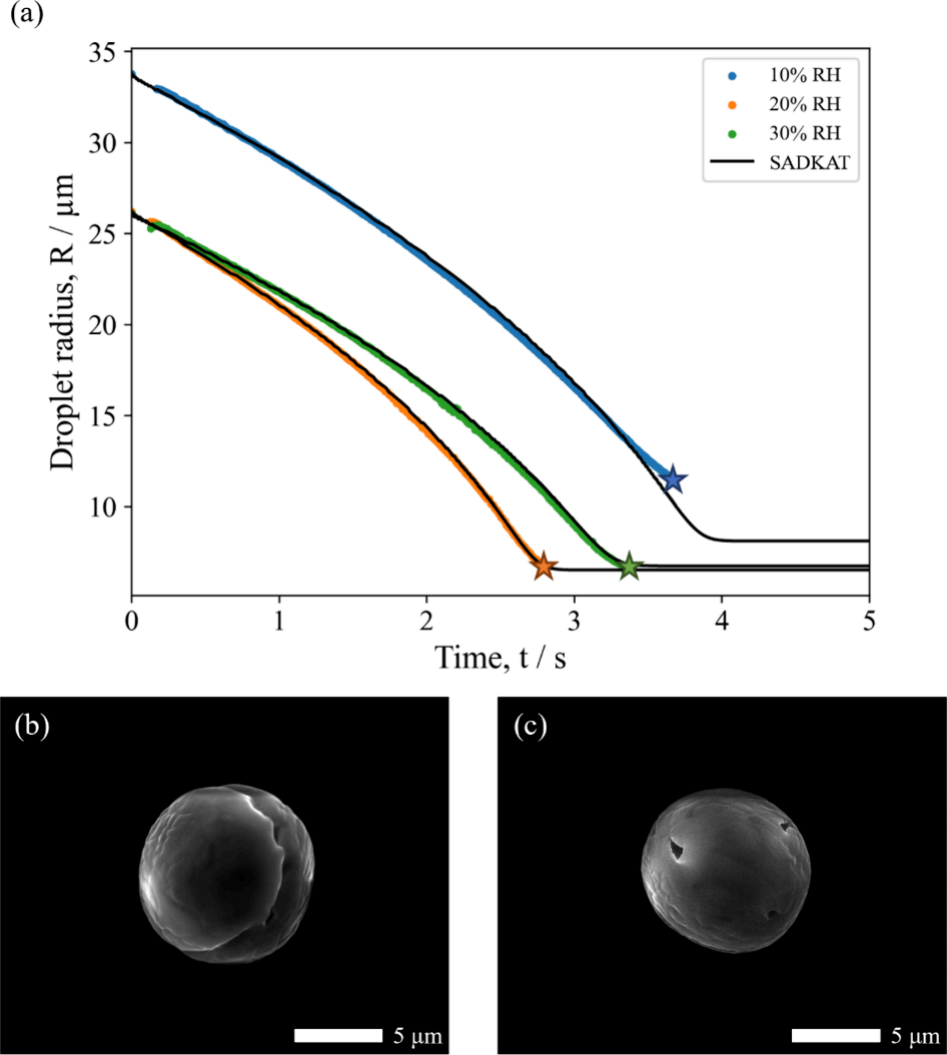
(a) Evaporation profiles for aqueous (NH_4_)_2_SO_4_ (0.02 MFS) droplets at varying RH and 293.2K compared with evaporation profiles modeled by SADKAT. Crystallization occurs at the time point indicated by a star, where the average *a*_w_ was calculated to be 0.424 ± 0.028% and the estimated dry particle size was 5.51 ± 0.01 μm. (b) SEM image of a (NH_4_)_2_SO_4_ microparticle dried at 0% RH, 294 K. (c) SEM image of a (NH_4_)_2_SO_4_ microparticle dried at 20% RH, 294 K.

For (NH_4_)_2_SO_4_ aerosol droplets, there have been investigations into the drying kinetics,^47^ thermodynamics,^48,49^ and the impact of thermodynamics on the aerosol morphology.^50,51^ However, there is no literature on the impact of drying kinetics on the morphology of the dried microparticles. Figure 3a shows the evaporation profiles of (NH_4_)_2_SO_4_ droplets at various RHs below the ERH. The close agreement of the EDB measurements with the SADKAT model provides validation for the drying kinetics shown. This plot also demonstrates SADKAT's ability to accurately model droplets of different sizes, with the droplet at 10% RH having a radius of ≈8 μm larger than those at 20 and 30% RH. The deviation of the EDB measurement and SADKAT model just before the crystallization point in the 10% RH evaporation profile is likely due to surface enrichment in the droplet, which SADKAT does not account for. The degree of surface enrichment increases with evaporation rate, which is why this phenomenon is not seen at the higher RHs.

SEM images of the resultant dried (NH_4_)_2_SO_4_ microparticles at different RHs are shown in Figure 3b,c. Unlike NaCl, the shape of the resultant (NH_4_)_2_SO_4_ microparticles appears to be independent of RH, with dense spheres forming at both 0 and 20% RH. Indeed, the circularity of particles formed at these two RHs is very similar (Figure 2d). From Table 1, however, it can be seen that the size of the microparticles is larger at 0% RH (5.13 ± 0.12 μm) than at 20% RH (4.87 ± 0.12 μm). This is likely a result of the increased evaporation rate increasing the degree of surface enrichment in the (NH_4_)_2_SO_4_ droplets, resulting in larger final microparticles at lower RHs. This supports the observation that the discrepancy in the EDB measurement versus the SADKAT model seen at 10% RH in Figure 3a is the result of surface enrichment.

**Table 1:** Average Dried Size of NaCl:(NH_4_)_2_SO_4_ Microparticles with Varying Mole Fraction (*X*_NaCl_) and RH

*X*_NaCl_	RH/%	microparticle size/μm
0.00	0	5.13 ± 0.12
0.00	20	4.87 ± 0.13
0.25	0	4.32 ± 0.12
0.25	20	3.63 ± 0.04
0.50	0	4.78 ± 0.08
0.75	0	4.69 ± 0.06
0.75	30	5.62 ± 0.09

### 3.2 Drying Kinetics, Morphology, and Composition of Aqueous NaCl:(NH_4_)_2_SO_4_ Aerosols

#### 3.2.1 NaCl:(NH_4_)_2_SO_4_ (*X*_NaCl_ = 0.25)

**Figure 4:**
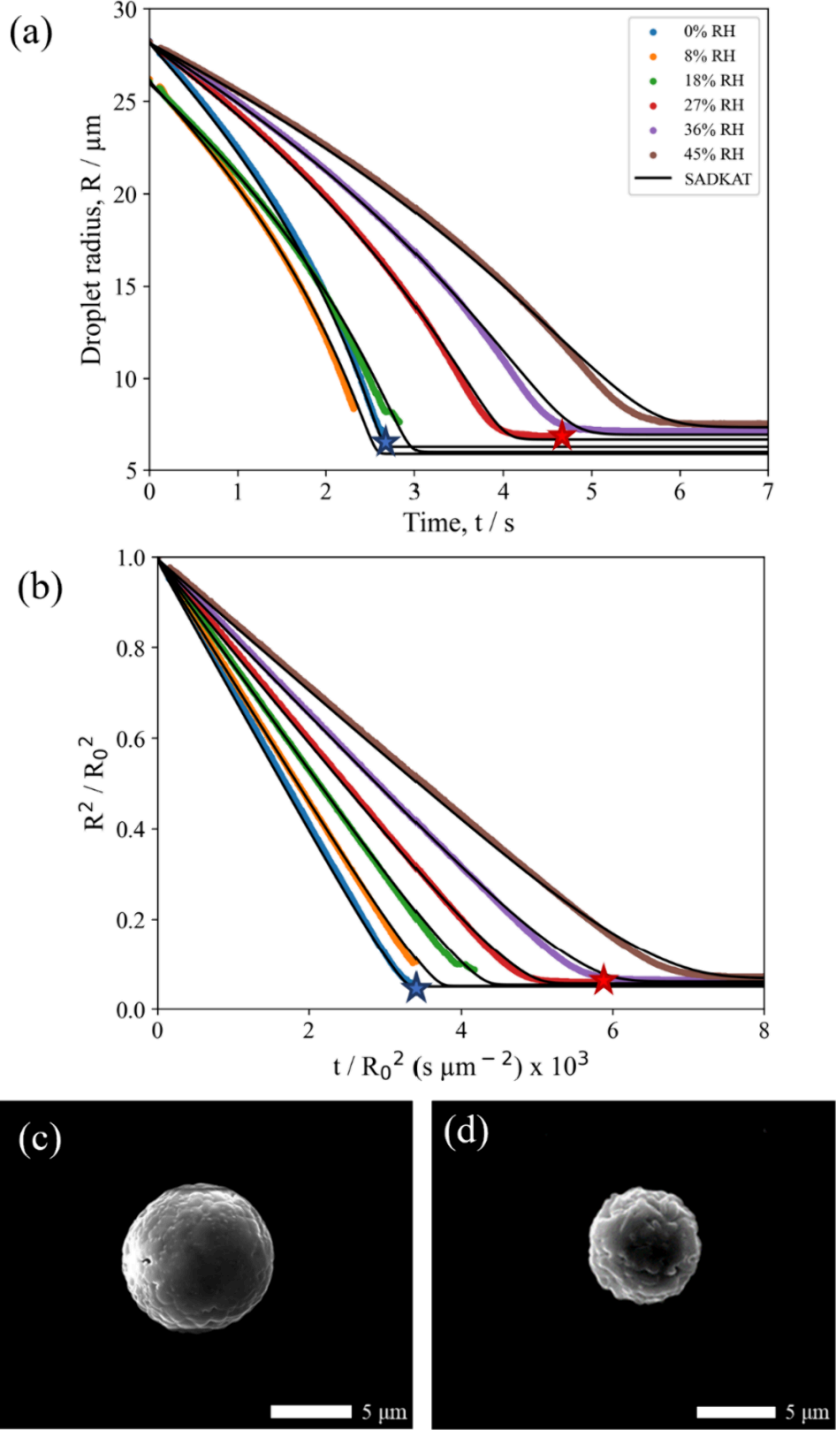
(a) Evaporation profiles for aqueous NaCl:(NH_4_)_2_SO_4_ (*X*_NaCl_ = 0.25, 0.02 MFS) droplets at varying RHs and 291.5 K compared with evaporation profiles modeled by SADKAT. The star points indicate the point of crystallization in the evaporation process, where the average *a*_w_ was calculated to be 0.360 ± 0.021 and the estimated dry particle size was 6.29 ± 0.01 μm. (b) Normalized radius squared plots for the data shown in panel a. (c) SEM image of a NaCl:(NH_4_)_2_SO_4_ microparticle dried at 0% RH, 294 K. (d) SEM image of a NaCl:(NH_4_)_2_SO_4_ microparticle dried at 20% RH, 294 K.

Figure 4a shows the evaporation profiles of NaCl:(NH_4_)_2_SO_4_ (*X*_NaCl_ = 0.25) droplets drying under various RHs. This is the first comparison between EDB measured evaporation profiles and those modeled using SADKAT for a mixed salt aerosol droplet. It is clear from this plot that the model and experimental data match well. Figure 4b gives a clearer picture of the drying kinetics by showing the droplet evaporation in terms of the normalized radius squared, where the gradient of the plot is then equal to the droplet evaporation rate. This figure clearly demonstrates that increased RH decreases the evaporation rate, an expected result in line with previous work.^18−20^ The lack of an identifiable crystallization event for the droplets evaporating at 8 and 18% RH is due to the instability of the droplets in the EDB, with each droplet lost from the trapping region before crystallization was observed. Clear and consistent crystallization was observed for droplets evaporating at 27% RH; droplets evaporating at lower RHs can also be expected to crystallize.

The impact of the drying kinetics on the NaCl:(NH_4_)_2_SO_4_ (*X*_NaCl_ = 0.25) microparticle morphologies is similar to that of pure (NH_4_)_2_SO_4_. The shape of the microparticles seems to be unaffected by changes in RH as the particles are spheres at both 0 (Figure 4c) and 30% (Figure 4d) RH, although the sizes of the microparticles are larger at the lower RH (Table 1). Unfortunately not enough particles were imaged to create a statistically relevant histogram for the “circularity analysis”, but the SEM images clearly show this qualitative trend. This indicates, as with the pure aqueous (NH_4_)_2_SO_4_ droplets, that the higher evaporation rates at the lower RH increase the degree of surface enrichment and lead to a larger dried particle. When these mixed salt droplets are dried, the dissociated ions in the aqueous phase (Na^+^, Cl^−^, NH_4_^+^, and SO_4_^2−^) can recombine to form NaCl and (NH_4_)_2_SO_4_, or they can form new salts such as NH_4_Cl or Na_2_SO_4_.^52^ The dried salt particles formed at thermodynamic equilibrium can be calculated using E-AIM.^45^ For the NaCl:(NH_4_)_2_SO_4_ (*X*_NaCl_ = 0.25) system, the thermodynamically stable composition is a 5/2/1 molar ratio of (NH_4_)_2_SO_4_/NH_4_Cl/Na_2_SO_4_. The large mole fraction of (NH_4_)_2_SO_4_ here correlates with the similarity seen between this mixed salt particle morphology and that of a pure (NH_4_)_2_SO_4_ microparticle.

**Figure 5:**
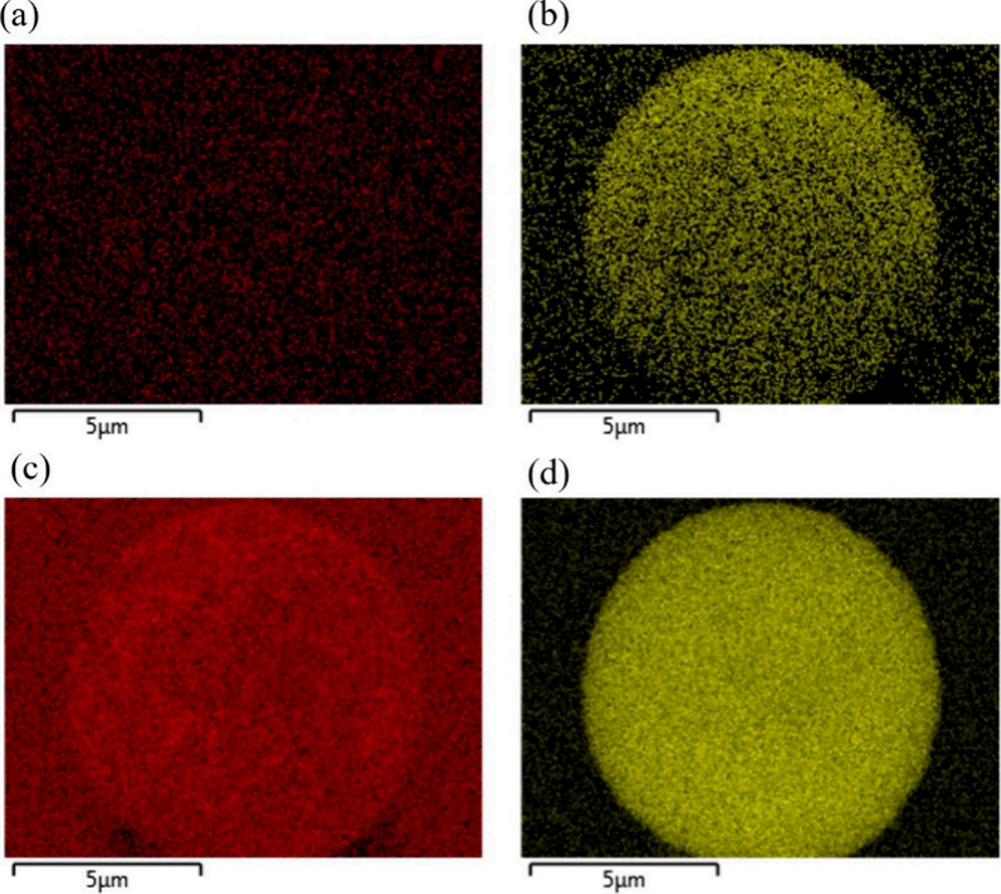
EDX mapping of a NaCl:(NH_4_)_2_SO_4_ (*X*_NaCl_ = 0.25, 0.02 MFS) microparticle dried at 0% RH and 294 K. Elements mapped are (a) chlorine, (b) nitrogen, (c) sodium, and (d) sulfur.

SEM-EDS analysis was performed on the dried microparticles to observe their dried composition (Figure 5). It is clear from Figure 5a that there was no chlorine remaining in the dried microparticle, indicating that there has been chlorine loss from the system, and the particle is composed of just (NH_4_)_2_SO_4_ and Na_2_SO_4_. It has been well documented that NH_4_Cl decomposes in aerosol particles, with Harrison et al. (1990) showing that the rate of degradation is on the scale of tens of minutes to hours.^53^ Chlorine can also be lost from aerosol droplets via a displacement reaction with H_2_SO_4_, which may be present in the mixed salts investigated here.^54,55^ Angle and Grassian (2023) showed that there was significant chloride depletion during the evaporation lifetime of water from a micron-sized aqueous droplet of NaCl/glycine/Na_2_SO_4_.^56^ Of these two mechanisms, it is more likely that chloride is lost through the degradation of ammonium chloride. If the mechanism was via the chloride displacement reaction, then a divergence between the EDB measured evaporation profiles and the SADKAT model would be expected in Figure 4a,b as the loss of chloride would be on the same scale as water evaporation from the droplet. The agreement between the model and measured data indicates that there was no loss of the inorganic components during the measurement window. The work by Tobon et al. (2021) supports this hypothesis as they performed Raman spectroscopy on dried NaCl:(NH_4_)_2_SO_4_ particles at a range of *X*_NaCl_ from 0.1 to 0.67. In each case, they detected the presence of NH_4_Cl after 30s.^52^ Additionally, E-AIM predicts the partial pressures NH_3_ and HCl to reach maximums of 1.48 × 10^−4^ and 6.09 × 10^−9^ atm during the drying process, supporting the assumption that volatilization is unimportant during the droplet drying process. However, between sample collection and SEM imaging, the glass slides containing the salt particles were all stored in a drying cabinet (Section 2.3). Storage at elevated temperatures (66 °C) for several days would facilitate the decomposition of the NH_4_Cl.

Contrary to the previous literature on the morphology of mixed salts, these particles appear to have a uniform/homogeneous composition. Previous works show that mixed salts result in heterogeneous particles with a core/shell structure.^29−31^ This difference could be due to differences in the RH/evaporation rate at which the salts have dried. In the work of Li et al. (2014), there are no drying kinetic measurements or listed drying RHs for the reported atomic maps for the NaCl/KCl system. It could also be due to the identity of the solvents evaporating from the droplets. Ge et al. (1996) reported the formation of heterogeneous particles for NaCl/KCl, KCl/KI, and (NH_4_)_2_SO_4_/NH_4_NO_3_, but these salts were dissolved in 50/50 (v/v) water/ethanol solution. These alcohol/water droplets will have higher evaporation rates when compared to a pure water droplet, and this likely influences the final particle morphology.

#### 3.2.2 NaCl:(NH_4_)_2_SO_4_ (*X*_NaCl_ = 0.50)

**Figure 6:**
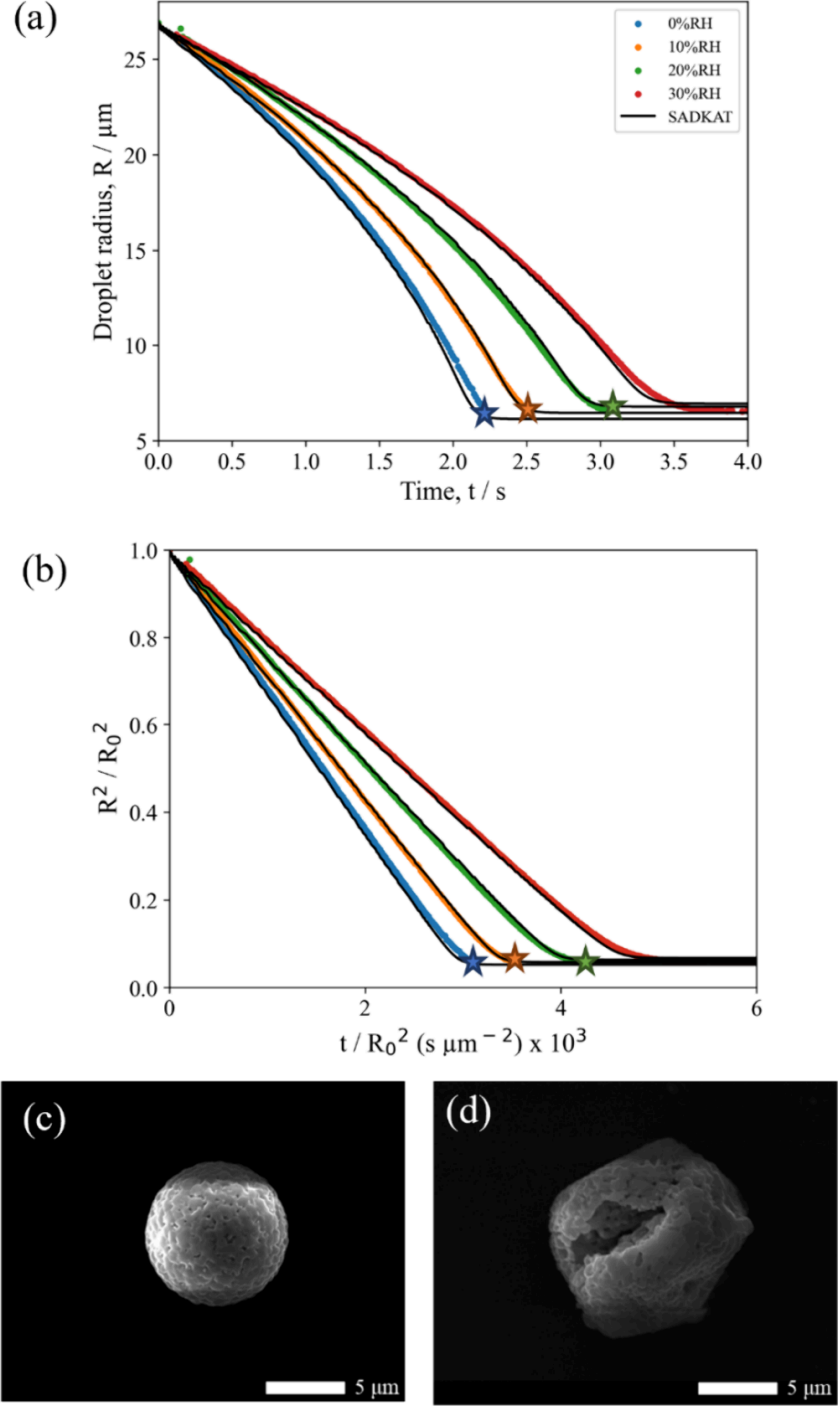
(a) Evaporation profiles for aqueous NaCl:(NH_4_)_2_SO_4_ (*X*_NaCl_ = 0.5, 0.02 MFS) droplets at varying RHs and 293.6 K compared with evaporation profiles modeled by SADKAT. The star points indicate the point of crystallization in the evaporation process, where the average *a*_w_ was calculated to be 0.356 ± 0.032 and the estimated dry particle size was 5.11 ± 0.04 μm. (b) Normalized radius squared plots for the data shown in panel a. (c) SEM image of a NaCl:(NH_4_)_2_SO_4_ microparticle dried at 0% RH, 294 K. (d) SEM image of a NaCl:(NH_4_)_2_SO_4_ microparticle dried at 20% RH, 294 K.

**Figure 7:**
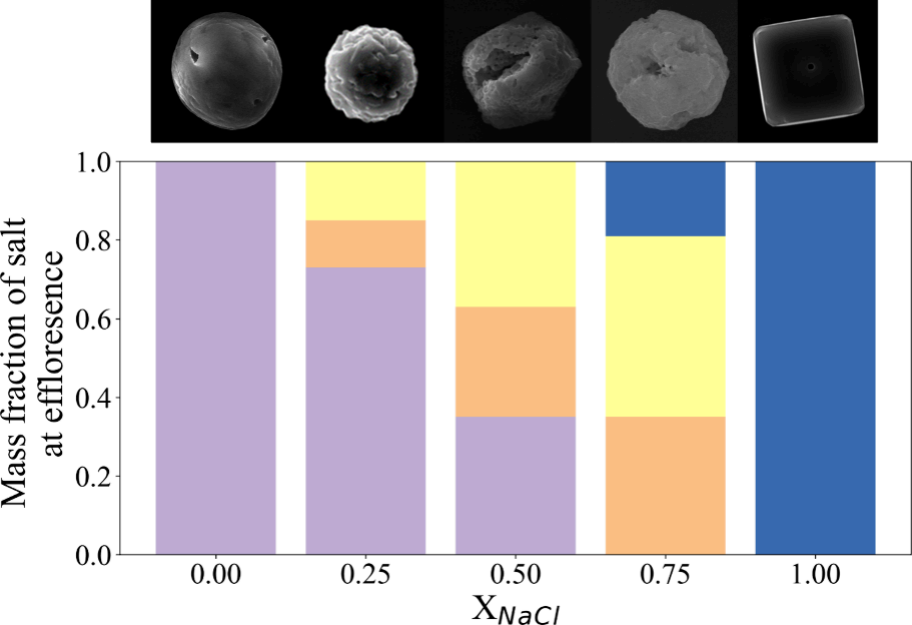
Mass fraction of the salts predicted by E-AIM to be present in the dried particle at the efflorescence point with SEM images for morphological comparison. Purple = (NH_4_)_2_SO_4_, orange = NH_4_Cl, yellow = Na_2_SO_4_, and blue = NaCl. The RH input for E-AIM was obtained by calculating the *a*_w_ from the evaporation profiles (see Figures 1, 2, 3, 5, and 7). The change in droplet size from the kinetic data allows for an MFS at the efflorescence point to be calculated; this can be related to an *a*_w_ using the “*a*_w_ vs MFS parametrizations” (Table S1).

Figure 6a,b shows the evaporation profiles of aqueous NaCl:(NH_4_)_2_SO_4_ (*X*_NaCl_ = 0.5) droplets drying under various RHs. As with the plots in Figure 4a,b, it is clear that there is good agreement between the experimental evaporation profiles and those modeled by SADKAT, further indicating that the model is able to accurately simulate evaporation profiles of droplets containing two involatile components. The impact of the drying kinetics on the NaCl:(NH_4_)_2_SO_4_ (*X*_NaCl_ = 0.5) dried microparticle morphology is shown in Figure 6c,d. Unlike the microparticles seen for the mixed *X*_NaCl_ = 0.25 system, the RH at which the droplets dried has a clear impact on the morphology of the resultant microparticle. Drying at 0% RH (Figure 6c) results in a spherical microparticle, whereas a slower evaporation rate results in a more angular particle containing clefts (Figure 6d). This behavior is consistent with the observations seen in the pure component salts and the mixed *X*_NaCl_ = 0.25 system and is supported by the plots of degree of circularity with changing RH (Figure 2c). Evaporation at low RH consistently leads to spherical microparticles due to the effect of high surface concentrations leading to multiple nucleation sites. At high RH, the influence of particle composition comes into play. As was discussed in Section 3.2.1, the morphology of the mixed *X*_NaCl_ = 0.25 microparticle resembles that of pure (NH_4_)_2_SO_4_. Given the large mass fraction of (NH_4_)_2_SO_4_ in the resultant microparticle (Figure 7), this observation is unsurprising. We can also see from Figure 7 that the ratios of salts in the resultant *X*_NaCl_ = 0.50 microparticles are relatively similar, but that the mass fraction of Na_2_SO_4_ is the largest. The morphology of pure Na_2_SO_4_ microparticles dried at 40% RH (Figure S1) looks similar to that of the mixed *X*_NaCl_ = 0.25 microparticles dried at 20% RH (Figure 6d). Both are particles with large clefts in the center, indicating that the concentration of the thermodynamically stable salts impacts the morphology at high RHs.

The predicted composition of the dried *X*_NaCl_ = 0.50 salt particles at thermodynamic equilibrium is 1/2/1 molar ratio of (NH_4_)_2_SO_4_/NH_4_Cl/Na_2_SO_4_. SEM-EDS analysis showed that, as with the dried *X*_NaCl_ = 0.25 salt particles, there was a complete loss of chlorine from the microparticles (Figure S2). As above, there was good agreement between the SADKAT model and measured EDB data, indicating that there was no loss of the inorganic components during the measurement window.

#### 3.2.3 NaCl:(NH4)2SO4 (*X*_NaCl_ = 0.75)

**Figure 8:**
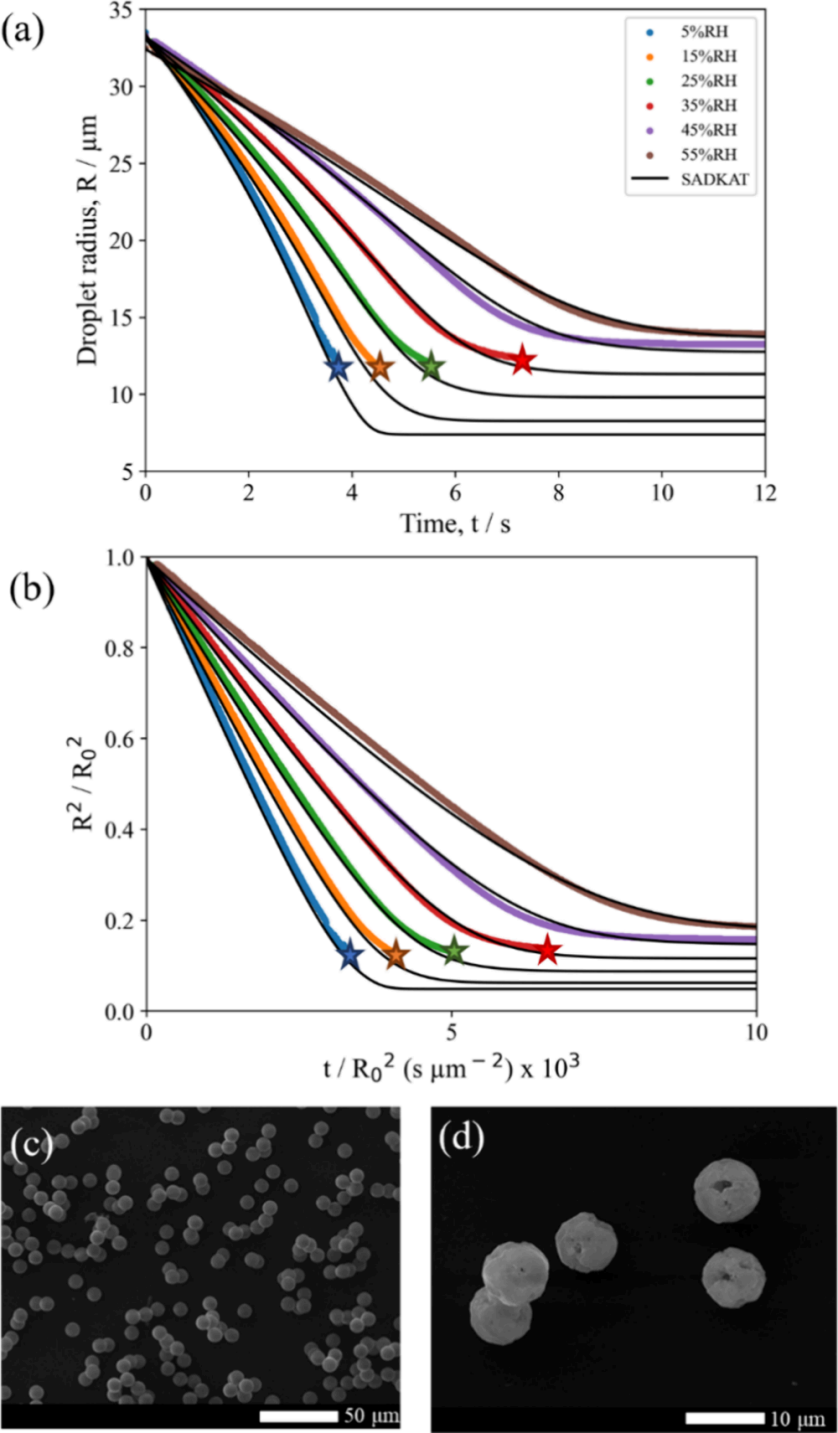
(a) Evaporation profiles for aqueous NaCl:(NH_4_)_2_SO_4_ (*X*_NaCl_ = 0.75, 0.02 MFS) droplets at varying RHs and 293.5 K compared with evaporation profiles modeled by SADKAT. The star points indicate the point of crystallization in the evaporation process, where the average *a*_w_ was calculated to be 0.415 ± 0.010 and the estimated dry particle size was 7.19 ± 0.07 μm. (b) Normalized radius squared plots for the data shown in panel a. (c) SEM image of NaCl:(NH_4_)_2_SO_4_ microparticles dried at 0% RH, 294 K. (d) SEM image of NaCl:(NH_4_)_2_SO_4_ microparticles dried at 30% RH, 294 K.

Figure 8a,b shows the evaporation profiles of aqueous NaCl:(NH_4_)_2_SO_4_ (*X*_NaCl_ = 0.75) droplets drying under various RHs. As with the other mixing ratios, there is good agreement between the modeled evaporation profiles and the EDB measurements. There is a slight divergence near the efflorescence points, likely due to the slow drift in the RH of the trapping chamber over time. Figure 8c,d shows SEM images of populations of dried NaCl:(NH_4_)_2_SO_4_ (*X*_NaCl_ = 0.75) microparticles. In line with the previous sections, at low RH, the particles form dense spherical microparticles due to the larger surface enrichment experienced at high evaporation rates. At 30% RH, the microparticles are still roughly spherical but are more segmented, with several holes and clefts. This observation is again supported by the circularity analysis (Figure 2b) and is consistent with the lower degree of surface enrichment expected at higher RH, as fewer nucleation sites result in particles with distinct sections.

As with the dried microparticles from the *X*_NaCl_ = 0.50 mixed salt solution at 20% RH, particles from the *X*_NaCl_ = 0.75 mixed salt solution at 30% RH have a similar morphology to that of a pure Na_2_SO_4_ microparticle dried from a solution droplet at 40% RH (Figure S1). From Figure 7 it can be seen that Na_2_SO_4_ makes up the largest composition of the dried NaCl:(NH_4_)_2_SO_4_ (*X*_NaCl_ = 0.75) aerosol, again supporting the observation that the concentration of the thermodynamically stable salts impacts the morphology at high RHs. Interestingly, the size of the resultant microparticles increases from 0 to 30% RH (Table 1), an opposite trend from the *X*_NaCl_ = 0.25 mixed aerosol. This could be due to the hydration of the Na_2_SO_4_ salt in the resultant microparticle, as higher relative humidities would lead to greater degrees of hydration and therefore larger particle size. This is supported by the E-AIM model of this system, where it is predicted to contain a hydrated Na_2_SO_4_.(NH_4_)_2_SO_4_.4H_2_O component between 71 and 76% RH.

SEM-EDS was performed to confirm the composition of the resultant particles. Figure S3 shows that chlorine, sodium, and sulfate were present at both 0 and 30% drying RHs, but not nitrogen. Given that the composition predicted by E-AIM was NH_4_Cl/Na_2_SO_4_/NaCl, this absence of nitrogen again indicates that NH_4_Cl has decomposed from the dried sample and also shows that the loss is independent of relative humidity. It can also be seen from these atomic maps that the compositions of the particles are homogeneous at both RHs. This supports the findings of Section 3.2.1 where it was shown that the NaCl:(NH_4_)_2_SO_4_ (*X*_NaCl_ = 0.25) particles were also homogeneous.

### 3.3 Drying Kinetics, Morphology, and Composition of Aqueous NaCl:NH_4_NO_3_ Aerosols

**Figure 9:**
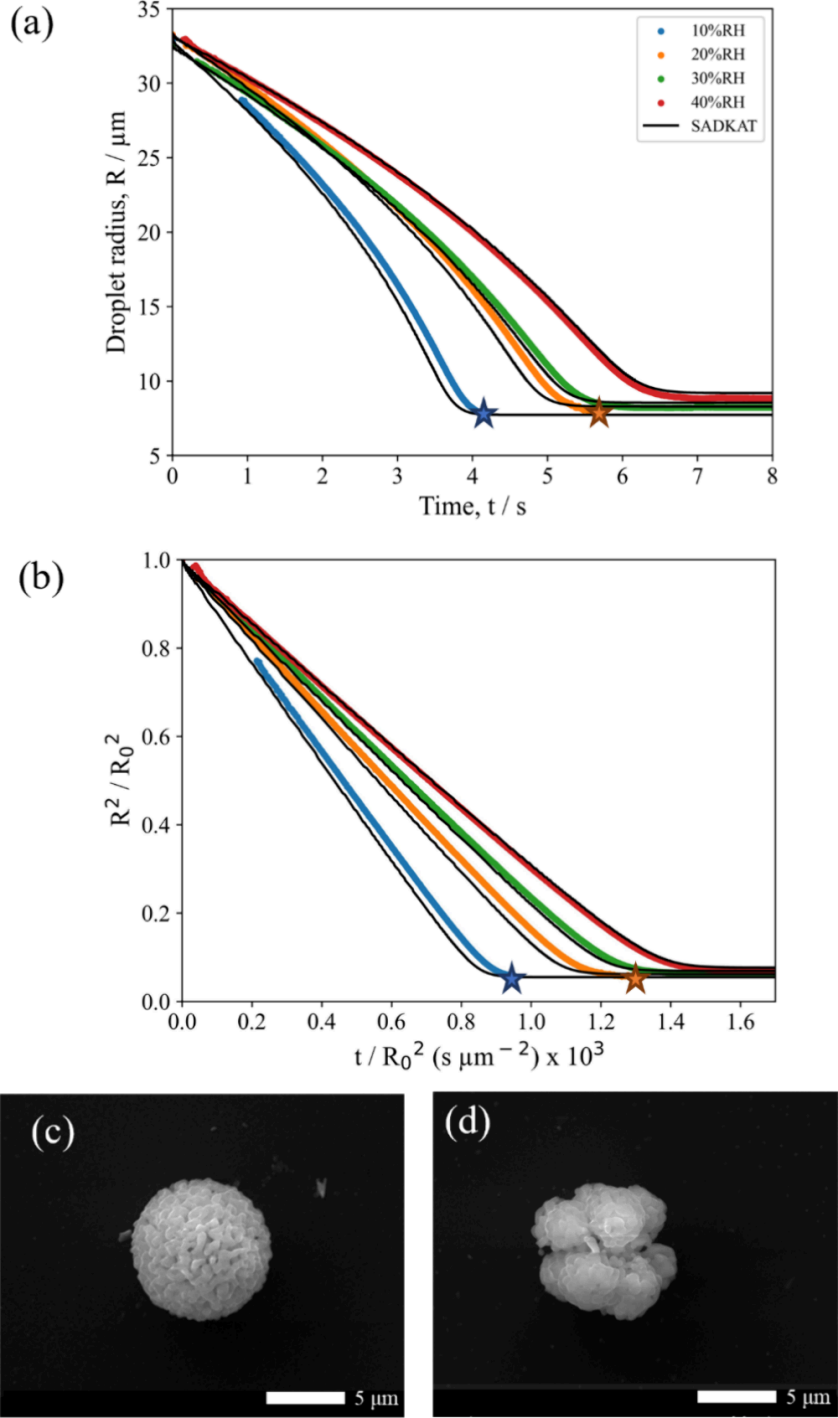
(a) Evaporation profiles for aqueous NaCl:NH_4_NO_3_ (*X*_NaCl_ = 0.50, 0.02 MFS) droplets at varying RHs and 293.4 K compared with evaporation profiles modeled by SADKAT. The star points indicate the point of crystallization in the evaporation process. (b) Normalized radius squared plots for the data shown in panel a. (c) SEM image of a NaCl:NH_4_NO_3_ microparticle dried at 0% RH, 294 K. (d) SEM image of a NaCl:NH_4_NO_3_ microparticle dried at 20% RH, 294 K.

A 1:1 molar ratio of NaCl:NH_4_NO_3_ was also investigated as an additional validation of the ability of the SADKAT model to simulate the evaporation profiles of mixed salt solution droplets. Figure 9a,b shows the evaporation profiles for NaCl:NH_4_NO_3_ droplets at various RHs. There is good agreement between the modeled and measured data sets, with the slight offsets between the profiles at 10 and 20% RH likely a function of slight differences between the predicted and true RH in the drying chamber.

Figure 9c,d shows SEM images of the resultant microparticles at 0 and 20% RH, respectively. The relationship between drying kinetics and morphology at 0% RH is consistent with the previous systems as the microparticles are spherical, indicative of a particle formed from a droplet with a large degree of surface enrichment where the high number of nucleation events preserves the droplet morphology. The particles formed at 20% RH are more segmented, having the appearance of several amalgamated spheres rather than a single sphere. This is consistent with the slower evaporation rates experienced by these aerosols as each of the subspheres indicates a nucleation event, showing a decreased degree of surface enrichment in comparison to the droplet dried at 0% RH.

**Figure 10:**
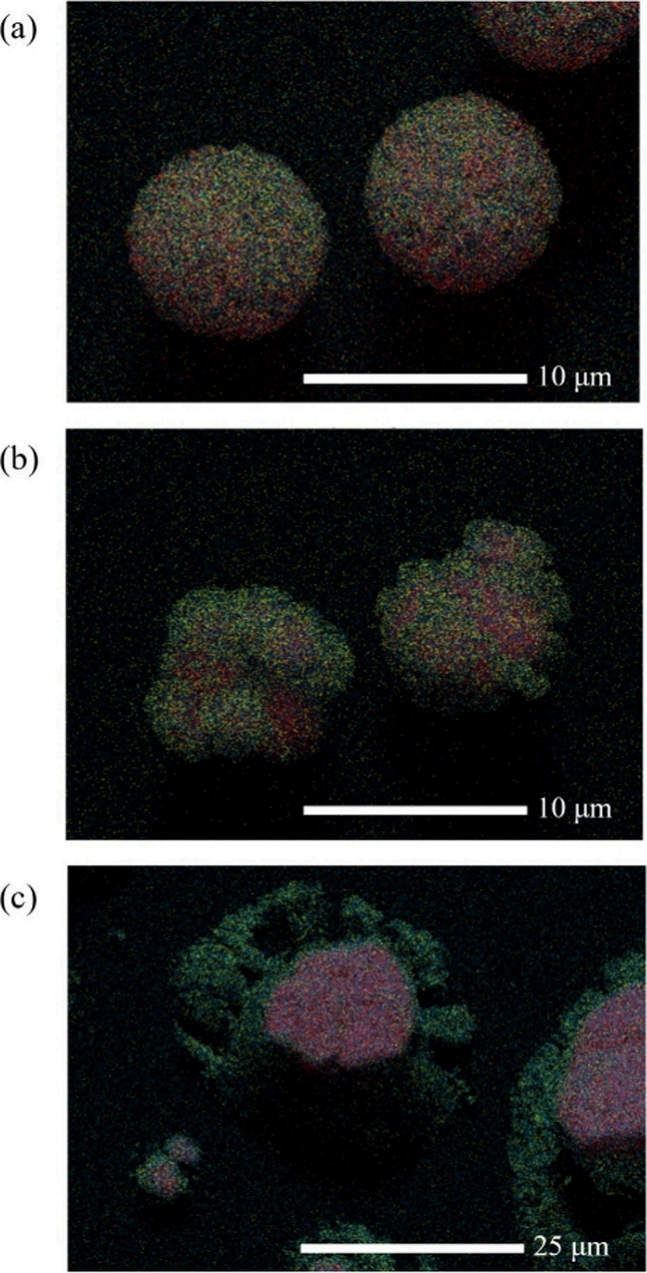
EDS mapping of NaCl:NH_4_NO_3_ (*X*_NaCl_ = 0.50, 0.02 MFS) microparticles dried at (a) 0% RH, (b) 20% RH, and (c) 40% RH. Elements mapped are chlorine in red, nitrogen in yellow, and sodium in blue.

The composition of the resultant microparticles is predicted by E-AIM to be a 1/1 molar ratio of NH_4_Cl/NaNO_3_. SEM-EDS atomic maps of droplets dried at various RHs can be seen in Figure 10. The droplets dried at 0% RH (Figure 10a) show a homogeneous mix of sodium, chlorine, and nitrogen. This makes it difficult to make any certain claims on the exact salts present in the particle. However, as the RH is increased, the compositions of the particles show an increasing heterogeneity. Figure 10b,c suggests that there is a NaCl core to the resultant microparticles surrounded by NaNO_3_. The absence of the ammonia ion in the EDS suggests that NH_4_Cl was formed but is degraded into NH_3(g)_ and HCl_(g)_ as in the NaCl:(NH_4_)_2_SO_4_ systems. The formation of NaCl salt, which was unpredicted by E-AIM, is likely due to the fact that the ERH of NaCl is higher than both NH_4_Cl and NaNO_3_.^57^ E-AIM is a thermodynamic model and does not account for the crystallization of salts, a kinetically determined process.

As has been discussed previously, the presence of heterogeneity in mixed salt particles has been well established in the literature, but the trends seen here seem to indicate that this heterogeneity arises when the particles are dried in an RH that is near to or above the ERH of one of the pure components but below the other. The ERHs of NaCl and NH_4_Cl are ≈47 and 45%, respectively.^31,57^ NaNO_3_ is unique in that there is not an agreed efflorescence point.^34^ Tang and Munkelwitz (1994)^58^ reported an ERH of 20−30% RH, but Hoffman et al. (2004)^59^ and Liu et al. (2008)^60^ did not observe any distinct efflorescence. Baldelli et al. (2016) suggested that this behavior is a consequence of increased viscosity slowing the nucleation kinetics.^27^ In either case, it is clear that the ERH of NaNO_3_ is much lower than that of NaCl and NH_4_Cl. NH_4_Cl has been lost from these particles and so cannot be commented on, but NaCl is clearly acting as a nucleation seed at 20 and 40% RH, with the NaNO_3_ being insufficiently supersaturated to homogeneously effloresce.

### 3.4 Drying Kinetics, Morphology, and Composition of Aqueous NaCl:CaCl_2_ Aerosols

**Figure 11:**
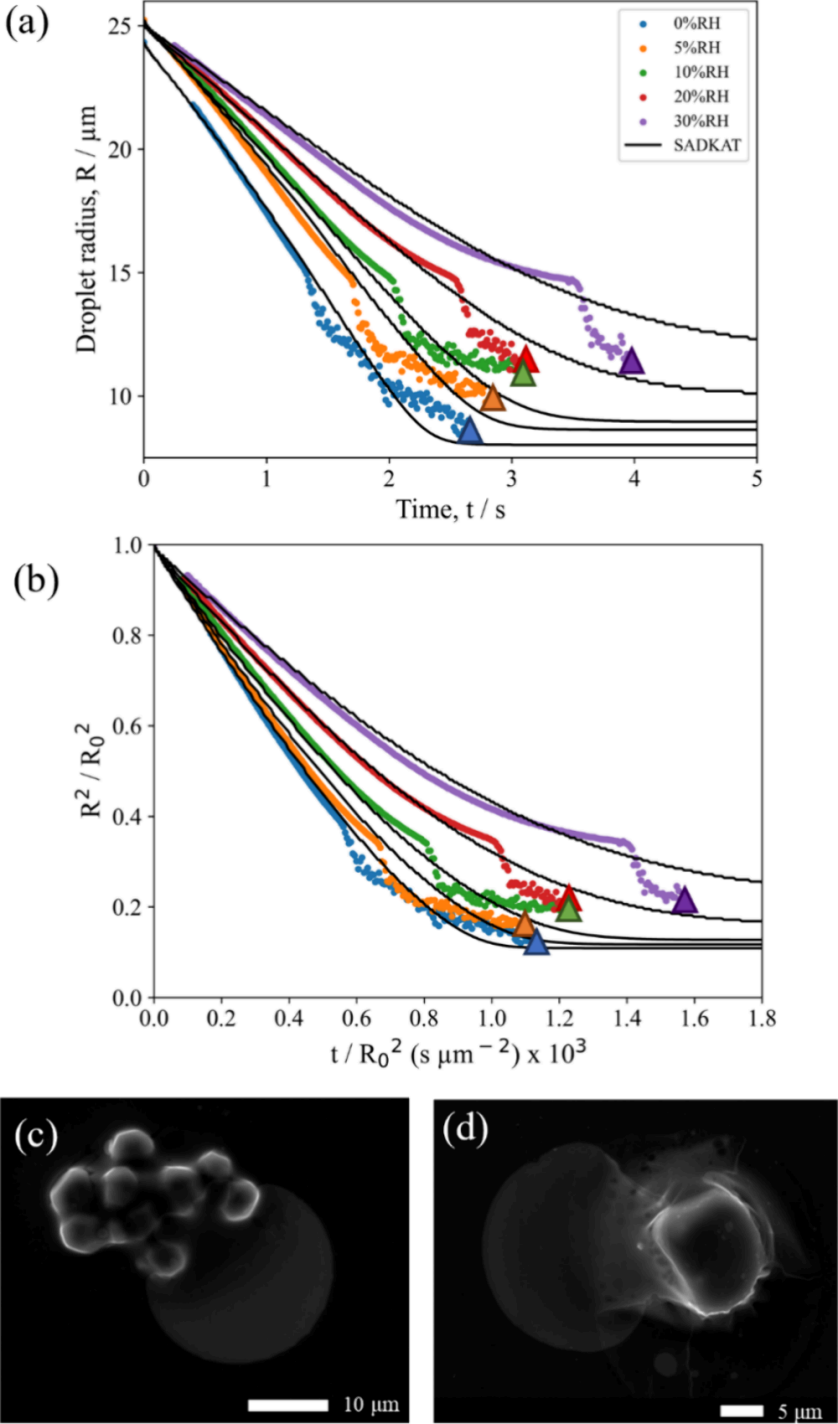
(a) Evaporation profiles for aqueous NaCl:CaCl_2_ (*X*_NaCl_ = 0.65, 0.1 MFS) droplets at varying RHs and 294.0 K compared with evaporation profiles modeled by SADKAT. The triangle points indicate the equilibration of the particle radius in the evaporation process. (b) Normalized radius squared plots for the data shown in panel a. (c) SEM image of a NaCl:CaCl_2_ microparticle dried at 0% RH, 294 K. (d) SEM image of a NaCl:CaCl_2_ microparticle dried at 40% RH, 294 K.

Figure 11a,b reports the evaporation profiles of NaCl:CaCl_2_ droplets (*X*_NaCl_ = 0.65) at various RHs. Again, there is good agreement between the measured evaporation profiles and those calculated by SADKAT. Unique to this aerosol system is that the particle size can be estimated and continues to decrease in radius after the efflorescence point, which is identifiable by a sudden decrease in radius. This implies that, although there has been a crystallization event, water continues to evaporate from the particle. Figure 11c,d shows SEM images of the resultant microparticles at 0 and 40% RH, respectively. In both cases, it seems that the particles were not fully dry when deposited on the glass slide, evidenced by the disc of salt surrounding the salt crystals. At both RHs, some salt has crystallized, but this has not triggered heterogeneous nucleation of any other salt phase. From the ERHs of NaCl and CaCl_2_ (47 and 2−4% RH, respectively),^30,61^ it is likely that the NaCl has homogeneously crystallized with Ca^2+^ and Cl^−^ ions remaining in the aqueous phase until after deposition. This is also indicated by the change in morphology of the salt crystal. At low RHs, the core has a multicrystal structure similar to that of pure NaCl droplets at low RH (Figure 1b). At high RH, a single crystal has formed, which is again similar to the behavior of aqueous NaCl droplets (Figure 1c). The evaporation profiles corroborate this, with the nucleation point of the NaCl clearly shown in Figure 11a,b, but the continued decrease in radius indicates that the CaCl_2_ component is still present as an aqueous solution and more slowly losing water. These findings are consistent with the observations in the previous sections where heterogeneous particle formation only seems to occur when the salts are dried near or above the salt components' pure ERH. In this case, a NaCl core is surrounded by an aqueous CaCl_2_ “shell” that does not homogeneously effloresce.

## 4 Conclusions

This work studied the effects of adding an additional salt to NaCl aerosol droplets with respect to the drying kinetics, dried particle morphology, and dried particle composition. RH influenced the drying kinetics of these droplets, in line with previous investigations, with the SADKAT model showing good agreement with all salt mixtures and at all RHs. Comparing evaporation profiles to the SEM images of particles dried at the same RH allowed for the evaluation of the impact of drying kinetics on the particle morphology. The impact of the high evaporation rate on the final particle morphology was in line with previous studies, with high surface enrichment and spherical particles being observed. At lower evaporation rates, the particle morphology is influenced by the salt with the highest concentration in the droplet at efflorescence. Furthermore, the composition observed in the dried particles of this study builds on the work done in efflorescence kinetics by showing that heterogeneous particles are formed when the ambient RH is similar to or above the ERH of one of the component salts in the droplet. If the ambient RH is lower than the ERH of both salts, then homogeneous salt particles are formed. In summary, the morphology and composition of the resultant particles in these mixed salt systems can be estimated using drying kinetics data, thermodynamic predictions of the salt composition, and the ERH of each component salt.

## Data Availability

Data are available at the University of Bristol data repository, data.bris, athttps://doi.org/10.5523/bris.1u33ng7oor6h229wt3j4tqsvki.

## Supporting Information Available

SEM image of a Na_2_SO_4_ droplet (0.02 MFS) dried at 0% RH and 294 K; SEM-EDS spectrum of a NaCl:(NH_4_)_2_SO_4_ (*X*_NaCl_ = 0.50) particle dried at 0% RH; SEM-EDS mapping of NaCl:(NH4)2SO4 (*X*_NaCl_ = 0.75, 0.02 MFS) microparticles dried at varying RHs and 294 K; table of parameters used for each insoluble component in the SADKAT model; and table of input parameters for the evaporation profiles modeled using SADKAT (PDF)
