# Supplementary material for: Evaporation Kinetics and Final Particle Morphology of Multicomponent Salt Solution Droplets
Source: J Phys Chem A. 2025 Jan 11;129(3):762–73. doi: 10.1021/acs.jpca.4c07439 (PMC11770748; doi:10.1021/acs.jpca.4c07439)
Supplement: Supplementary file 1 — jp4c07439_si_001.pdf [file jp4c07439_si_001.pdf]

Evaporation Kinetics and Final Particle Morphology of multi-component Salt Solution Droplets

Barnaby E. A. Miles, Emily Winter, Shaira Mirembe, Daniel Hardy, Lukesh K. Mahato, Rachael E. H. Miles and Jonathan P. Reid*

School of Chemistry, University of Bristol, Bristol, BS8 1TS, UK
*Corresponding author (j.p.reid@bristol.ac.uk).

Figure S1. SEM image of an Na_2SO_4 particle dried at 45% RH and 294 K.

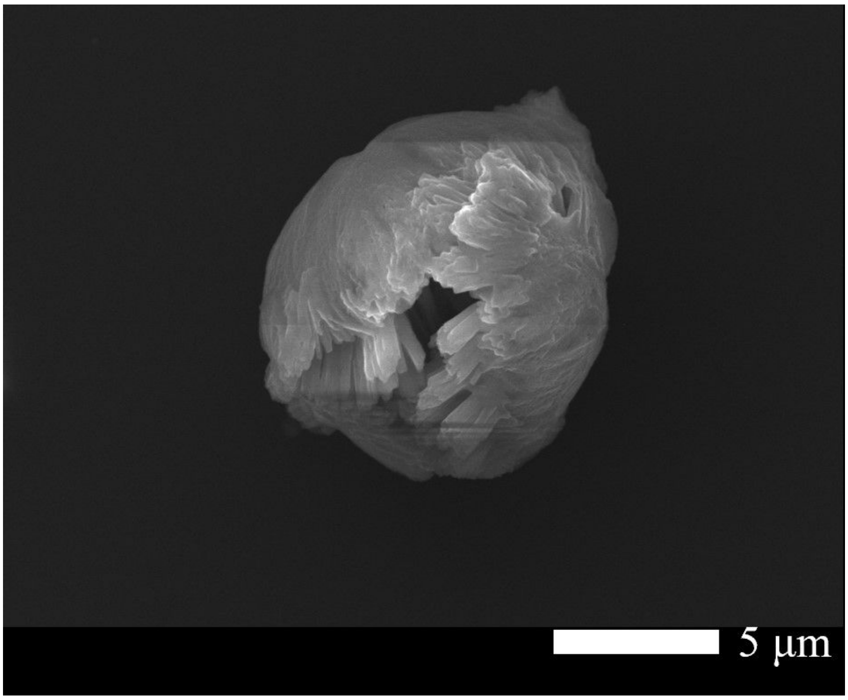

Figure S2. SEM-EDS spectrum of (a) a $NaCl:(NH_4)_2SO_4$ (X_{NaCl} = 0.50) particle dried at 0% RH and 294 K, (b) the glass slide the particles were collected on.

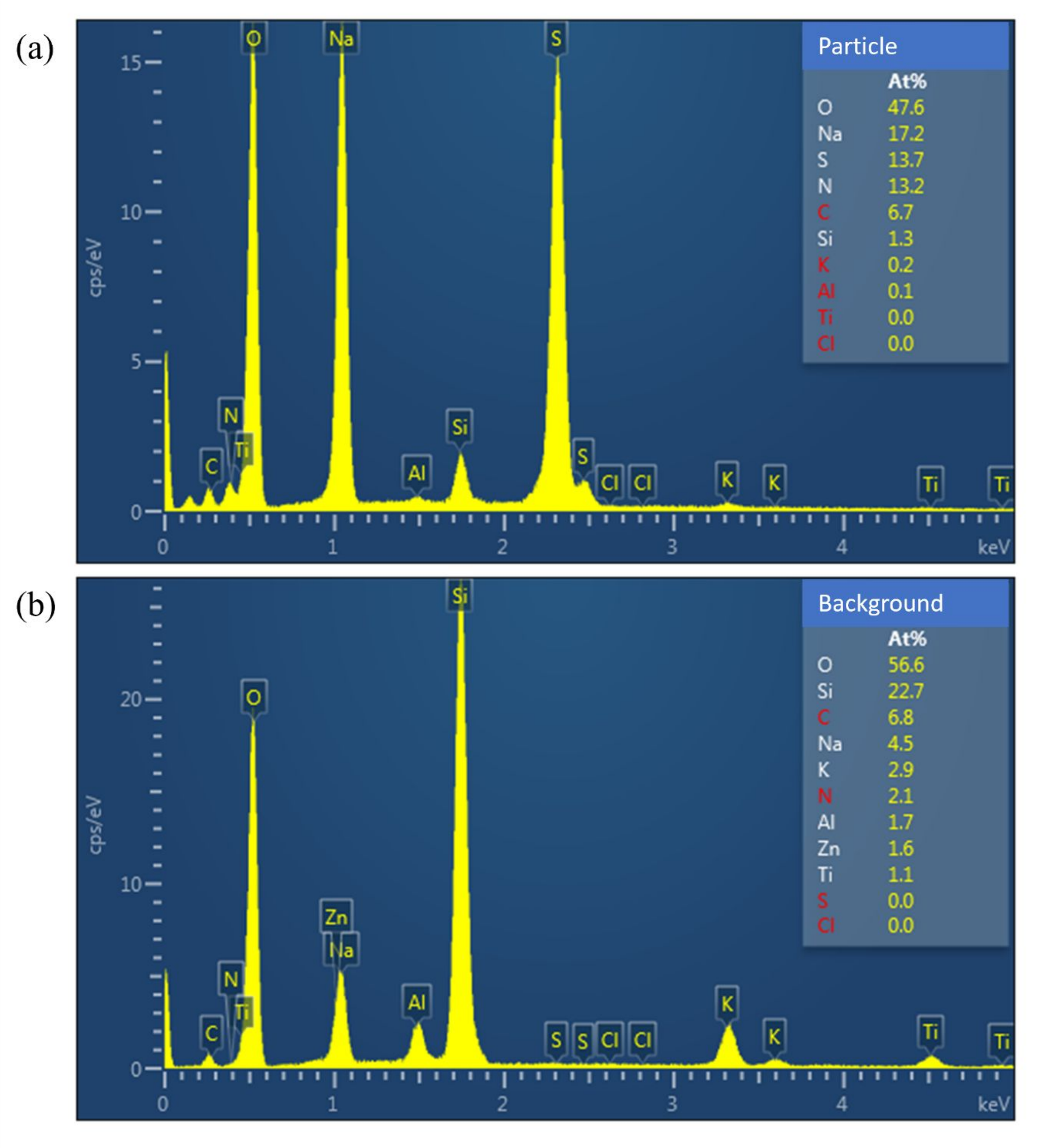

Figure S3. EDS mapping of $NaCl:(NH_4)_2SO_4$ (X_{NaCl} = 0.75, 0.02 MFS) microparticles dried at varying RHs and 294 K.

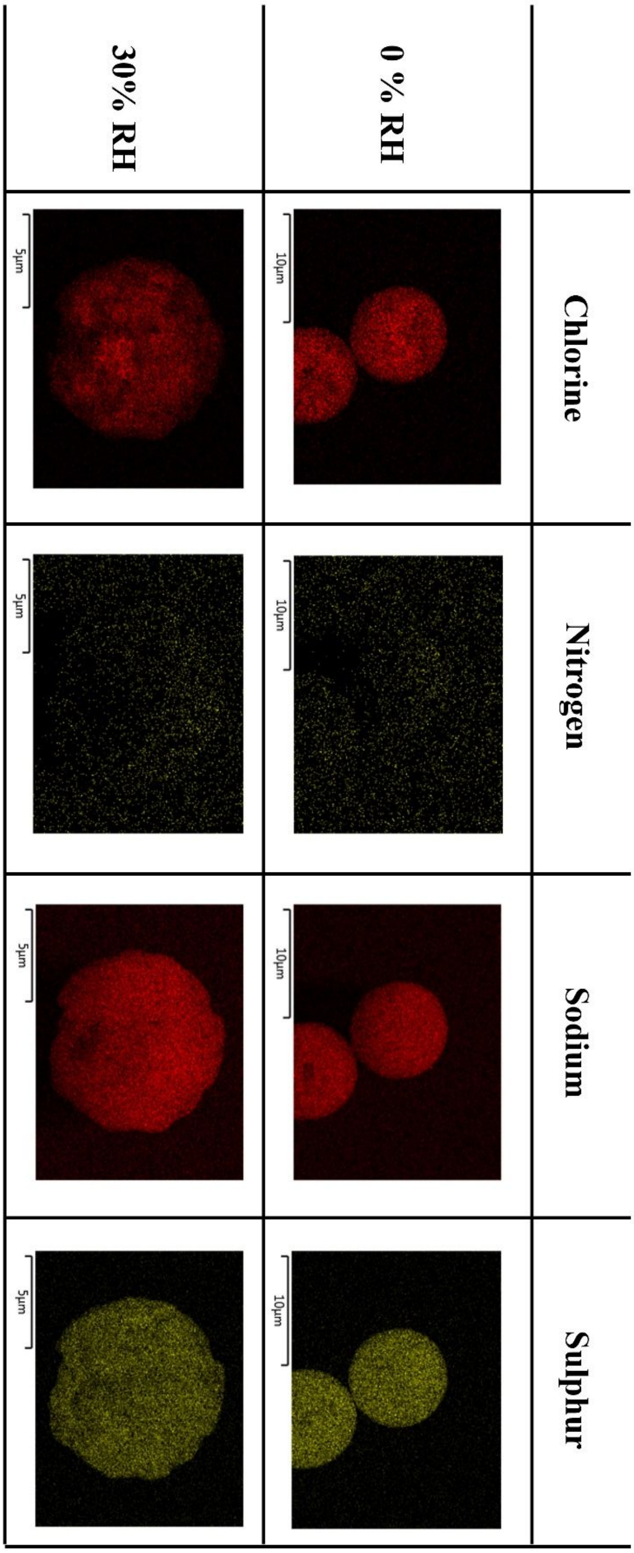

Table S1: Parameters used for each insoluble component in the SADKAT model.

component	molar mass / g mol^{-1}	solute density / Kg m^{-3}	density vs MFS parametrisation	a_w vs MFS parametrisation
NaCl	58.44	2170	998.2 , -55.3, 1326.7, -2131.1, 2895.9, -940.6	48.53, -158.04, 186.59, -93.89, 19.29, -3.00, -0.48, 1.00
$(NH_4)_2SO_4$	132.14	1770	1033.4, -298.5, 1307.2, -1576.2, 1074.6	-0.27, 0.36, -1.00, -0.08, 1.00
NaCl:$(NH_4)_2SO_4$ (X_{NaCl} = 0.25)	113.15	1812	998.2, -75.4, 977.3, -1197.9, 1110.6	-15.16, 44.10,-50.80, 28.07, -7.10, -0.12, 1.00
NaCl:$(NH_4)_2SO_4$ (X_{NaCl} = 0.50)	95.86	1871	996.78, 264.73, -676.52, 1286.3	-3.97, 9.01, -8.60, 4.26, -1.71, 1.00
NaCl:$(NH_4)_2SO_4$ (X_{NaCl} = 0.75)	76.88	1973	998, -116.6, 1304, -1822.3, 1610	0.06, -1.55, 3.14, -2.62, 1.00
NaCl:NH_4NO_3 (X_{NaCl} = 0.50)	69.24	1720	1006.3, -44.5, -64.0, 6420.6, -29860.2, 69135.5, -84917.4, 53233.9, -13383.1	189.84, -787.23, 1354.51, -1256.21, 683.90, -221.59, 40.37, -4.24, -0.35, 1.00
NaCl:$CaCl_2$ (X_{NaCl} = 0.65)	76.56	2155	998, 94.1, -309.6, 2833.8, -3945.9, 2484.6	-0.99, 2.18, -2.06, 1.00

Table S2: Input Parameters for the evaporation profiles modelled using SADKAT. 'Temperature / K' was used for both the droplet and ambient temperature.

component	RH / %	temperature / K	initial droplet diameter / µm	mass fraction solute
NaCl	10	293.0	26.22	0.02
	20		26.34	
	30		26.58	
	40		26.63	
$(NH_4)_2SO_4$	10	293.2	26.22	0.02
	20		26.10	
	30		26.01	
$NaCl:(NH_4)_2SO_4$ ($X_{NaCl} = 0.25$)	0	291.5	26.19	0.02
	8		26.05	
	18		28.21	
	27		28.22	
	36		28.22	
	49		28.26	
$NaCl:(NH_4)_2SO_4$ ($X_{NaCl} = 0.50$)	0	293.6	26.80	0.02
	10		26.81	
	20		26.89	
	30		26.70	
$NaCl:(NH_4)_2SO_4$ ($X_{NaCl} = 0.75$)	5	293.5	33.06	0.02
	15		33.49	
	25		33.20	
	35		33.20	
	45		33.24	
	55		33.28	
$NaCl:NH_4NO_3$ ($X_{NaCl} = 0.50$)	10	293.4	32.88	0.02
	20		33.25	
	30		32.49	
	40		33.17	
$NaCl:CaCl_2$ ($X_{NaCl} = 0.65$)	8	294.0	24.33	0.10
	14		25.23	
	24		25.10	
	32		25.09	
	42		25.06	
